# The therapeutic use of the dog in Spain: a review from a historical and cross-cultural perspective of a change in the human-dog relationship

**DOI:** 10.1186/s13002-017-0175-6

**Published:** 2017-08-23

**Authors:** José Ramón Vallejo, Dídac Santos-Fita, José Antonio González

**Affiliations:** 1 Área de Didáctica de las Ciencias Experimentales, Equipo de Historia de la Ciencia y Antropología de la Salud, Facultad de Educación, Universidad de Extremadura, E-06006 Badajoz, Spain; 2 Centro de Investigación en Ciencias Biológicas Aplicadas, Universidad Autónoma del Estado de México, Avda. Instituto Literario 100, Colonia Centro, CP 50000 Toluca, Estado de México Mexico; 3 Grupo de Investigación de Recursos Etnobiológicos del Duero-Douro (GRIRED), Facultad de Biología, Universidad de Salamanca, E-37071 Salamanca, Spain

**Keywords:** Dog, Dog-derived products, Zootherapy, Ethnomedicine, Spain

## Abstract

In Spain, studies about traditional knowledge related to biodiversity have focused on vascular plants. For this reason, our review concentrates on the identification and inventory of zootherapeutic resources, particularly those involving the dog (*Canis lupus familiaris* Linnaeus, 1758) throughout the twentieth century to the present. A qualitative systematic review in the fields of ethnomedicine, ethnozoology and folklore was made. Automated searches in the most important databases and digital libraries were performed. All related works were examined thoroughly and information was obtained from 55 documentary sources. We have listed a total of 63 remedies to treat and/or prevent 49 human illnesses and conditions. In 20 of the documented reports the whole animal was used and the use of pups was recommended in 12 cases. Saliva was the healing element in 10 remedies, and faeces were the therapeutic basis for nine, while bitch’s milk was for seven of them. Skin, fur and meat were next in significance. Currently, healing remedies based on the use of the dog are not part of Spanish ethnomedicine and considering them so would be ahistorical. Indeed, the custom of allowing a dog to lick one’s wounds to assist in their healing and cicatrisation has survived in only a few groups of people. However, we can state that the ethnomedical use of the dog exists and has been transferred to “animal-assisted therapy”.

## Background

Broad international consensus exists on the need to preserve, protect, research and promote the biocultural heritage derived from traditional knowledge (TK) [1–3]. In Spain, the study and preservation of TK has been covered under legislation, both local and national, since the middle of the 1980s. This type of knowledge related to biodiversity resulted in the publication of the two stages of the *Spanish Inventory of Traditional Knowledge related to Biodiversity* (IECTB, for its acronym in Spanish) which, in compliance with the Law 42/2007 and the Royal Decree 556/2011, have so far been implemented (http://www.mapama.gob.es/es/biodiversidad/temas/inventarios-nacionales/inventario-espanol-de-los-conocimientos-tradicionales/inventario_esp_conocimientos_tradicionales.aspx). Although this Spanish legislation acknowledges the need to know, conserve and promote traditional practices of broad interest for biodiversity, the publications focus mainly on the TK relating to vascular plants. Therefore, in parallel and independently, our work is part of a project aimed at the identification, inventory and cataloguing of the Spanish zoocultural heritage and, more specifically, of the zootherapeutic resources [4–9].

Initially, the main objective of these works was to resolve problems of taxonomy and to identify wild animal species and taxa used in Spanish ethnomedicine, but given their cultural and anthropological importance, we have started to study the medical uses of domestic animals [10, 11].

The inventory and documentation of remedies based on the dog (*Canis lupus familiaris* Linnaeus, 1758) can be valuable for the understanding of the relationship of human communities (and their TK) with the domestic animals in twentieth century Spain. So, for example, some points of the philosophical-humoral basis of zootherapeutic knowledge could be clarified, together with its historic origin and other elements of symbolic thought in the rural environment, which has been more closely involved with these species rather than wild ones since the middle of the last century.

Dogs’ hearing and sense of smell are very well developed, the nose being their main sensory organ. In some cases their innate abilities can be used, while in others they have to be trained. The large majority of dogs are kept as pets, although there are many ways in which dogs can help humans (livestock guardian dogs, hunting dogs, guide dogs, assistance dogs, etc.). One of the current uses of this animal species can be considered as a “medicinal response”, which is the increasing function of assistance dogs in Europe and North America. They are trained to react on epileptic seizure (“seizure response dogs”) [12–14] or to identify patterns in skin and breath odours from their owners during a hypoglycaemic episode (“diabetes alert dogs”) [15, 16]. Another very interesting current use, that is gaining wider acceptance, is known as “animal-assisted therapy” (AAT), where the dog is a species much in demand for interacting with people with affective, emotional and cognitive needs, or those who are socially deprived. Not only are they used in healthcare, but also in gerontology and penitentiary institutions [17–20]. It should be noted that AAT is also known in Spain, as well as in other developed countries, under the name of “zootherapy”; although that is a broader concept and, in the context of ethnobiology, it refers to the medicinal use of animals and animal-derived products to treat illnesses and health conditions. Furthermore, it includes popular practices such as “magical prophylaxis” such as amulets and transference rituals [21, 22].

Without a doubt, the local knowledge regarding the treatment and prevention of disease forms part of the collective memory of any human community. The use of animal substances must be understood from a cultural point of view, since medical systems are organised within cultural systems [23–26]. A great variety of animals, both wild and domestic, form part of the pharmacopoeia of many regions around the world.

Zootherapy comes from ancient history and it is widely disseminated, which led Marques [27] to formulate a “zootherapeutic universality hypothesis”, according to which all human societies with medical systems employ remedies based on animals [21]. Specifically, the medical use of parts and derivative products of dogs (fat, hair, faeces, licking, etc.) was discussed already by Carl Linnaeus in his dissertation *Cynographia* [28], and is well-known from both European folk medicine [29–31] and officinal medicine. Dog-based remedies are mentioned in many European pharmacopoeias in the nineteenth [32] and probably the twentieth century as well. For example, *Album Graecum* is a term that was used by apothecaries to refer to dry white dog faeces that were especially rich in phosphate as a result of feeding dogs a bone-rich diet. This product has been used since at least the sixteenth century as an important ingredient in the preparation of ointments or liniments for the treatment of quinsy [33, 34] or, in the form of a poultice, as a septic drug to dry up hard tumours [28, 35]. Schurig, in chapter XIV of his work *Chylologia historico-medica* (1725), entitled *De Stercoribus Brutorum*, wrote that the one considered best, *Album Graecum*, came from white dogs, dogs qualified as “those of a more healthy nature” [36].

The objectives of this study were therefore: (1) to gather and document the traditional remedies based on the use of the dog as part of twentieth century Spanish ethnomedicine, and (2) to make a contribution that, together with the existing work on other animal species, allows an assessment to be made of the Spanish and European ethnozoological heritage in the medium term.

## Methods

The search for documentary sources to carry out this review of therapeutic products derived from dogs was based on national and international databases. The research was directed towards areas or disciplines related to folk medicine, such as social anthropology, ethnography, folklore studies, medical anthropology, the history of medicine and, of course, ethnobiology. The key words used concern the area of knowledge or discipline and the animal taxon, in this case the dog. Starting from the documentary sources found, other non-indexed works were used, and so it was possible to study an index that was difficult to access. Most of the documentary sources were found within the national context from the databases of the information system of the Spanish Research Council (Spanish acronym: CSIC) –ICYT (Science and Technology), ISOC (Social Sciences and Humanities) and IME (Biomedicine)–, the Ph.D. Theses TESEO database, the bibliographic web Dialnet, Google Scholar and the catalogue of Public State Libraries (BPE). However, international databases and digital libraries including the ISI Web of Science, Scopus, Anthropology Plus and JSTOR III – Arts & Sciences were also searched, corroborating the poor dissemination of Spanish and European ethnozoology.

The remedies obtained were ordered according to the chapters set out in the International Statistical Classification of Diseases and Related Health Problems 10th Revision, ICD-10 (http://apps.who.int/classifications/icd10/browse/2010/en). This categorisation system is very useful for clinicians, as their criteria are the guidelines for establishing diagnoses, but, logically, they do not coincide with the popular conception of a disease. For example, diseases, ailments and disorders exist that are hard to classify in conventional medical categories, frequently referred to as “cultural illnesses” [37]. However, this classification at a higher category level (the categories are known as “chapters”) is similar to other ethnographic or ethnobiological type classifications that emphasise the *ethic* point of view. On the other hand, these chapters are very broad since they are based on large groups of diseases related to organic systems or very general aetiologies (see the sections in “Traditional medicinal practices”). Logically, this taxonomic system creates increasingly specific classification groups in order to separate the diagnosis (subjective) from the signs (objective), and thus arrive at a biomedical treatment with levels of precision that we do not use. However, the use of this classification aids professionals in their approach in the field of biomedicine and enables TK to be standardised in a society such as that of Spain. Therefore, this classification at a higher chapter level enables popular diseases to be standardised in a simple way, as well as differentiated; it can also help readers unfamiliar with ethnobiology or ethnomedicine to approach the subject of ethnomedical studies.

## Results

### Documentary sources

Documentary sources were selected for ethnobiological reasons, especially considering those works that provided data from local informants using the ethnographic method. During the analysis of the works found, work was observed where a commendable compilation of remedies was carried out, but the materials and the method used were not specified, nor was any discussion sought, and sometimes bibliographical and personal data were included, with some ambiguity. It should be noted that in Spain there is no tradition of ethnozoology, and few studies from a social context perspective have been developed that address folk medicine and include zootherapeutic remedies [38–41]. For this reason, the inclusion criterion for a document in the bibliographical review was based on the existence of coherence between the native category or some anthropological evidence in the context of Spanish folk medicine and the ethnozoological studies carried out in Spain to date.

After carrying out a general analysis of the documents found and their bibliographic references, 55 documentary sources were selected. As for the type of these sources, we obtained use-reports from 22 journal papers, most of them (20) published in journals in the field of folklore and ethnography, and 29 books, 21 directly related to the study of ethnomedicine in a particular geographical area, and eight concerning superstition, folklore or ethnobotany. We also obtained data from four theses (three Ph.D. theses and one degree thesis), one of which belongs to the field of ethnobotany.

Regarding the publication year, we obtained data in studies published over the past 16 years, namely from 17 works published between 2000 and 2009 and another five published between 2010 and 2016. We also include information collected in nine works from the 1990s, 10 from the 1980s, four from the 1970s, six from the period of 1940 to 1958, and another four from the early twentieth century (between 1902 and 1927). Only 15 of 46 documented empirical remedies were collected in documentary sources published over the last 7 years (2010–2016). Among those recent works, in a very few cases the authors used the present verbal form. No magical remedies or transference rituals are still practised today. Only the wound healing activity of dog licking can be considered as currently in use.

### Infectious diseases

In the Alto Aragón region, pulmonary tuberculosis was treated by giving the patient a broth to drink made from newborn puppies. It was essential that the patient should not know the origin of the broth, or else the remedy would not be effective [42]. In San Sebastián (Guipúzcoa), in the middle of the last century, the remedy consisted of placing on the chest of the tuberculous patient a puppy slit open from one end to the other, which would be kept there for 12 h. It was said that on removal it would emit a foul odour “because it was removing the humours”, and that if the patient did not get better, after a few hours, the treatment had to be repeated by applying another puppy [43, 44]. This practice also found very wide popular acceptance in the Autonomous Community of Extremadura [45].

To combat tuberculous meningitis, in a rural area close to San Sebastián, a renowned healer used to prepare a broth by cooking six newborn puppies in a large pot, a concoction that the patient had to take several times a day [43, 46].

By magical transference of disease, it was claimed in Spain that diphtheria (popularly known as *garrotillo*) could be treated by applying to the neck of the sick child the recently removed testicles of a dog [47]. In the Ripollés district (Catalonia) dog excrement was applied to the neck as an antidiphtheric poultice [48].

To treat whooping cough in the provinces of Salamanca and Cáceres it was recommended to drink the milk of female dogs [41, 49, 50]. Transference rituals have been documented for the treatment of this infectious disease in Extremadura. In the north of the province of Cáceres a sick child would spit on a piece of bread and immediately throw it to a dog, which would acquire the disease on eating it. In Fregenal de la Sierra (Badajoz) they used to sew a hair of the sufferer into a piece of meat that they gave to a dog to eat. Only if the animal coughed after ingesting it would the disease be confirmed as having been transferred [45].

When a person was bitten by a rabid dog, in order to prevent any contagion of the rabies, in Oliva de la Frontera (Badajoz) three hairs from that dog would be placed on the area of the bite [51]. In the Canary Islands they used to burn four hairs from the tail of the dog and apply the resulting ash to the wound [52]. In Castile-La Mancha hair from the rabid dog was fried in olive oil and this oil was rubbed into the wound [53, 54].

Another documented preventive practice was to place on the bite area an *uña de San Milano*, the name in various villages around Badajoz given to the fifth claw that exists in some breeds of dog have on their hind paws [51]. In Galicia the belief still persists there that the mere possession one of these extra claws, which are still known by the name of *unlla de San Eleuterio*, is a guarantee of protection against rabies [55]. An old Spanish superstition already documented at the end of the nineteenth century [30] supports the idea that a dog with these extra claws is always protected against rabies.

To eliminate warts, in Lanzarote (Canary Islands) a dog was allowed to lick the affected area [52] and in San Vicente de Alcántara (Badajoz) the sperm of a dog was applied to the warts [56]. The removal of warts in Extremadura was also achieved by transference rituals; these involved putting a grain of salt on to a crust of bread and giving this to a dog to eat, which in this way would acquire the skin lesions. In Cedillo (Cáceres), they made sure that the result was better if the “bait” was given to the dog on the feast day of St. Roch, 16 August [56].

To combat tertian and quartan fever (malaria) in the provinces of Cáceres and Salamanca they used to carry out strange therapeutic transference rituals. In Guijo de Granadilla (Cáceres) the affected individual kneaded a cake with olive oil, which was then placed in the armpit. The cake impregnated with the exudation of the sick person was given to a dog to eat, which then acquired the fevers [57]. In Fuenteliante (Salamanca) the toenails were cut, and the pieces placed on to a piece of bread that was then given to a dog to eat [58]. In a similar way, at the other end of the country, in Reus (Tarragona), to transfer a patient’s fevers to a dog, which had to be black, it was given a piece of bread to eat into which the patient’s nail clippings had previously been inserted [59].

### Neoplasms

In order to combat cancer, in Abadiano (Vizcaya) at the start of the previous century it was recommended to eat the meat of a puppy [44]. As Iribarren already indicated [60], in the early decades of the twentieth century people in general had a macroscopic idea about cancer; they believed that it involved a voracious species of crab (the crab of the sign of the Zodiac), and what they used to do was simply to feed it as many pieces of raw meat as it would eat so that it would not torment the patient.

In cases of skin cancer, at the end of the nineteenth and start of the twentieth century in Galicia, people would place the powder obtained from burning the head of a rabid dog on to the ulcer to treat it [61], while in Fuentes de León (Badajoz) they would tip on to the ulcer the ash obtained from burning its skin [51].

### Diseases of the blood and nutritional conditions

In Doñana (Andalusia) people still recall the belief that a concentrated broth made from litters of puppies could be used to cure anaemia. The animals were cooked on a low heat until they were completely soft, and a thick white broth was obtained and given to the patient for three consecutive days [62].

To combat rickets (infantile osteomalacia) in the provinces of Cáceres and Salamanca it was recommended to give bitch’s milk to sick children [41, 49, 63]. However, and although it may seem even more distasteful, the most commonly used remedy against this childhood condition, which is the result of poor nutrition, was to administer the broth obtained from cooking some puppies and/or to consume their meat. This practice was documented in areas as far apart as Asturias [64, 65], Tarragona [59], Badajoz [57, 63] or Murcia [66].

In the Sierra de Segura (Albacete) dog faeces were left in the open air overnight and then boiled. This water was filtered and given to malnourished children to drink, as a reconstituent tonic [53, 54].

### Behavioural disorders and diseases of the nervous system

At the beginning of the twentieth century in Galisteo (Cáceres), when children had *penterre*, meaning that they remained for a long time without breathing and were very red from having a tantrum, they were given fried puppy meat to eat [58].

In Alburquerque (Badajoz) it was recommended to give bitch’s milk to combat chronic alcoholism [57].

Killing a dog, skinning it, eviscerating it and leaving it out in the open air for a day, to then cook the meat and eat it, was the remedy used in Villarino de los Aires (Salamanca) in cases of hemiplegia [41].

### Diseases of the eye and ear

Regarding cataracts, the Augustinian priest César Morán wrote at the beginning of the twentieth century: “…it is good to wear in the ears, like earrings, strips of skin taken from a dog that was rabid, and black; if the cataract is in the left eye, the strip should be placed on the right ear, and vice versa”. This author mentions that this remedy was collected from Dehesa de Coquilla (Salamanca) [58].

In Líjar (Almería), instilling a thimbleful of bitch’s milk into the ear was considered the best remedy to alleviate the pain of earache in children with otitis media [67].

### Diseases of the circulatory and respiratory systems

Dog’s saliva was used in Valencia del Ventoso (Badajoz) as an anti-haemorrhoidal medication [68].

For bronchitis and colds in Valencia de Alcántara (Cáceres) the consumption of bitch’s milk was recommended [50]. In the Sierra de Segura (Albacete) a tisane prepared from dried figs (*Ficus carica* L.), ears of maize (*Zea mays* L.), a shed snakeskin and dog excrement, all sweetened with honey, was administered to treat colds [53, 54].

For a sore throat in the Ripollés district (Catalonia) dog excrement was applied directly to the neck [48]. In the same way as described above for the treatment of pulmonary tuberculosis, in San Sebastián the remedy for treating pneumonia consisted of placing a puppy slit open one end to the other on the chest of the patient for 12 h [43, 44]. In contrast, in Ripollés a poultice of dog excrement was applied as a remedy for pneumonia [48].

To treat asthma in Fuerteventura (Canary Islands) a “puppy-dog stew” was prepared with water, salt and puppies (*bardinos*, a breed of dog native to the island). It was a strong broth that was administered to asthmatics, and the curative effects were surprisingly positive [69].

### Diseases of the digestive system

**Fig. 1:**
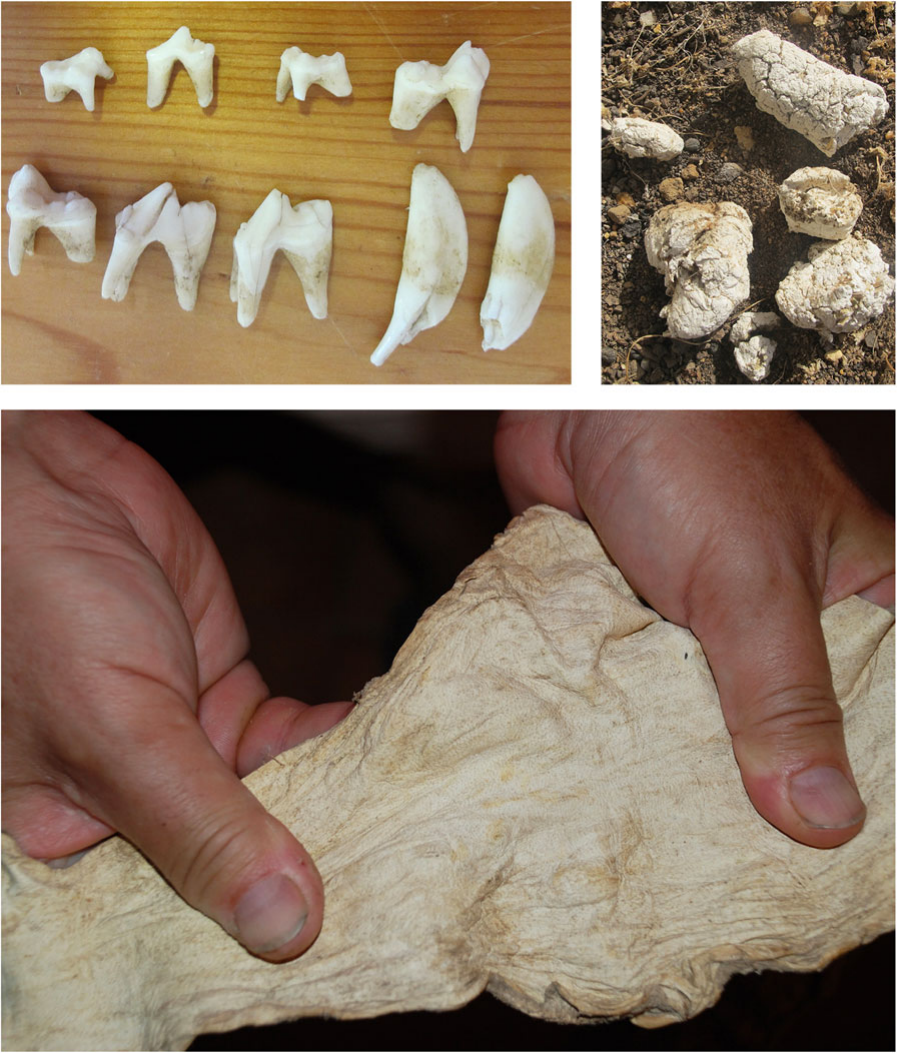
Examples of products used that were derived from dogs in Spanish ethnomedicine: various pieces of tooth, dry white faeces and a piece of tanned dog skin (photos by J. R. Vallejo and J. A. González)

Dogs’ teeth were among the amulets that were hung next to the heart of young child in the province of Salamanca to help with teething, and to encourage the growth of strong teeth [38, 41] (Fig. 1).

At the start of the twentieth century, a very popular transfer ritual to get rid of aphthous mouth ulcers (stomatitis), that were known as *lliras* (also locally as *liras* or *lirias*), was implemented in Asturias. With slight variations at local level, the ritual consisted of moistening nine pieces of bread with saliva, one each day, and going to give these to a dog to eat, saying: *Toma, can, / lliras y pan* (lit. “Take away, dog, / ulcers and bread”) [64, 65, 70]; or else, it consisted of smearing these nine pieces of bread with lard, going up with a dog towards a boundary stone and, once there, the patient would pretend to pull the ulcers out of his or her mouth and place them on the stone, then give the bread to the dog to eat, saying: *Lliras che quito, / nel marco las poño. / Toma, can, / lliras y pan* (lit. “Away you go, ulcers, / on the boundary stone I place you. / Take here, dog, / ulcers and bread”) [64, 65, 71, 72].

Also at the start of the twentieth century a curious custom still survived in Aldeanueva del Camino (Cáceres) for treating hernias in children (umbilical and inguinal), coinciding with the time of the feast of St. John. The child with the hernia had to hold the ears of a white dog that had recently died; the dog was then passed three times over the child, who was lying flat on the ground. The hernia would heal as the dog, hanging from a tree, rotted away [73].

To stop diarrhoea, in Bretún (Soria) they used to cook dried dog faeces and drink the resulting broth [74].

### Diseases of the skin and musculoskeletal system

To avoid the occurrence of hangnails around the fingernails, in San Vicente de Alcántara (Badajoz) they used to recommend rubbing the fingernails when two dogs mating were seen [56].

Dog saliva was regarded as the best remedy for chapped lips among the inhabitants of Holguera (Cáceres) and Zafra and Peloche (Badajoz) [51].

Against the symptomatic skin eruptions of urticaria, in Santa Cruz de Paniagua, El Bronco and Palomero (Cáceres) people used to draw the sign of the cross repeatedly over the affected area, using a sprig of mint (*Mentha* spp.) steeped in egg white beaten with red wine and dog’s saliva [75].

In Madroñera (Cáceres) it was believed that on the face, chest and sides of an infant suckling its mother, if she was pregnant, very painful spots would appear, which would only disappear if the child was administered some broth made from a dog that had recently given birth or from a newborn puppy [76].

To relieve the intense pain typical of sciatica, in the mountain villages of Álava the affected leg would be wrapped in a strip of dog skin tanned [46, 77] (see Fig. 1).

In order to prevent nocturnal leg cramps, in Navarra and Álava people used to bind the calves in straps made from dog leather [44, 60].

### Postpartum period

In women who were starting to breastfeed and whose nipples had changed shape, in order to obtain the correct nipple shape, the mother was given a puppy to suckle. This was a common practice in the first half of the twentieth century in Doñana (Andalusia) [62] and the Basque Country [44, 46]. In Bermeo (Vizcaya), once the task was finished, the animal was sacrificed because it was generally believed that it was for this reason that it would acquire rabies [78].

In Moraleja and Casas de Don Gómez (Cáceres) guide dogs would lick the fissures and sore areas on the breasts of the women who were breastfeeding because the saliva was considered to be curative and an effective remedy against such sores and excoriations [79].

Mastitis, also called mammitis, is the inflammation of breast tissue usually due to infection. To combat it, in the province of Soria they would give a newborn puppy to the affected mother to suckle [74, 80]. In Extremadura, the use of dogs in the treatment of mastitis is also known, “to remove the milk from the diseased breast”, and data exists about its cultural transmission to colonised villages, for example, from Fuente de Cantos to Guadiana del Caudillo (Badajoz) [40].

Milk stasis occurs when a milk duct is blocked and cannot drain properly. This may affect only a part of the breast and is not associated with any infection. Its treatment involved draining the breasts, emptying the milk. For this the most common practice in Navarra [44, 81], Segovia [82], Treviño –Burgos– [83], Soria [74], the Cerdanya district –Catalonia– [84], La Alcarria district –Guadalajara– [85] or the Basque Country [44] was to give a mother a puppy to suckle, preferably newborn.

Similarly, in order to drain breasts with excess milk in the Basque Country and Navarra the practice of using a puppy was frequently used [44, 78, 86, 87].

In Torrejoncillo (Cáceres) it is recorded that women with insufficient milk were made to drink bitch’s milk to try and increase the milk production quickly. In other places in Extremadura it used to be said that it was sufficient for the woman to eat the leftovers (food remaining) of a female dog that was suckling [76].

The problem of stopping breast-feeding (weaning) was solved in the Basque Country by the procedure of smearing the breasts with disgusting or bitter substances. The list of materials used was abundant: paprika (*Capsicum annuum* L.), bitter cheese and even dog excrement [86].

### Symptoms, signs and abnormal clinical and laboratory findings

In Arcos de Jalón (Soria) to prevent nosebleeds (epistaxis) the nose used to be blocked with dog faeces [74].

To combat jaundice in the provinces of Alicante, Valencia and Murcia, rituals were carried out to transfer the illness to a dog. In many villages it was a custom to urinate on a piece of bread and then give it to a dog to eat [88]. In the Campo de Cartagena district (Murcia) they used to mix the urine of the patient together with bran. They would use the first urine of the day. They made small balls which they gave to a black dog to eat each day for 9 days. The gradual decline of the animal was associated with the cure of the patient [66]. In Benimarfull and Muro de Alcoy (Alicante) lambs’ meat was boiled in the patient’s urine, a “stew” that was fed to a dog for three consecutive days [88].

### Injuries and other consequences of external causes

Even today, giving or offering a dog superficial wounds (cuts, chafing, etc.) to lick is common practice in Spain. According to informants from Salamanca, “what the dog licks heals quickly”, “the wound heals faster”, “the wound heals up better and much sooner”; and then “its saliva is better than ours”, “its saliva is a disinfectant”, “its saliva is very good for healing, cleaning and disinfecting wounds” and “its tongue is very good” [41].

**Fig. 2:**
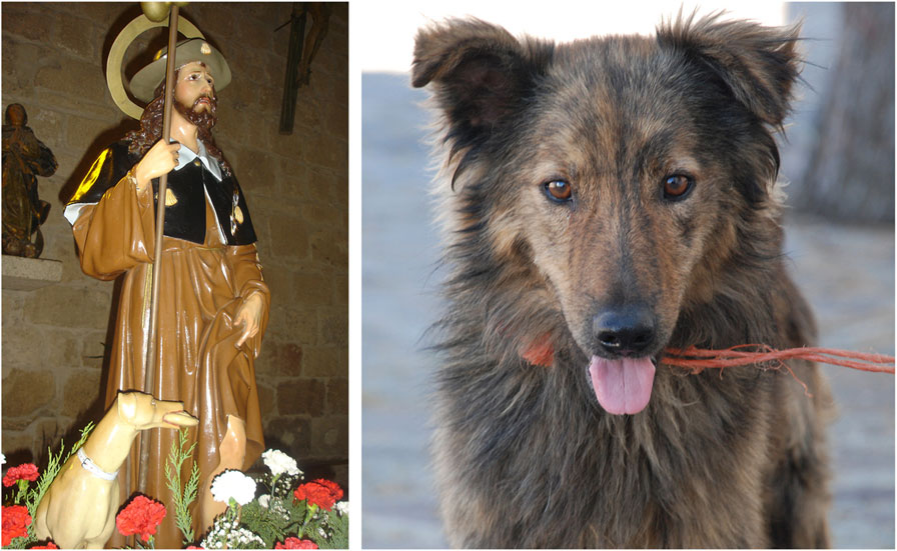
Image of St. Roch and panting dog (photos by J. A. González)

This practice probably resulted from the observation of how these animals looked after their wounds by licking them. This is a practice closely associated with pastoralism, as some key informants put it: “I’ve always heard shepherds say it”. It is also a remedy clearly associated with popular religion. They used to say that “dogs healed the wounds of Our Lord, that is why we shepherds, when we have a wound we offer the area to the dog to lick”, we were told by a shepherd from Almendra (Salamanca). In villages whose patron saint is St. Roch this is a very deeply rooted belief among the inhabitants. We believe that through the typical iconography of the saint, showing his wounds caused by the plague on his legs (the left one being shown most frequently) and attended by a dog (Fig. 2), that they are saying to us: “St. Roch’s dog did it to him” [41].

To stem the blood flow and to heal any type of wound, cut or sore (especially if it is infected) in the Basque Country [44], Navarra [60], Soria [74, 80] or Extremadura [57, 68, 79] the healing virtues attributed to dog licking have been considered extremely effective.

In a very different manner, in Ubierna (Burgos) for the treatment of infected wounds an ointment used to be applied made by frying a newborn puppy. The fat thus obtained was inserted into the urinary bladder of a pig, once it had solidified and was ready for use [89].

In Aldeanueva de Figueroa (Salamanca) to cure chilblains on the feet, dogs were used to lick them. In order to trick the animal they would put pork fat on to their feet [41].

### Animal bites and stings

The practice of giving the affected area to a dog to lick was used in the province of Salamanca in cases of bites and stings from harmful animals. In Morasverdes the affected area was presented for licking in cases of the most common insect bites and stings (wasps, bees, horseflies, etc.) and in Guijuelo against viper bites (*Vipera latastei* Boscá, 1878), for which a fairly deep cut was previously made with a razor [41].

In the Canary Islands, against the painful bite of the European black widow spider (*Latrodectus tredecimguttatus* Rossi, 1790), which in rare cases is considered fatal, the remedy commonly prescribed up until the middle of the last century was to take powdered human excrement. That found on roads, already dry, was preferred. Failing that, dog faeces were used, provided they were dry and had already turned a whitish colour [52] (see Fig. 1).

When someone was bitten by a dog he or she had to catch the animal and cut or pull a few hairs from its tail. These hairs were fried in a little olive oil and placed on the wound, previously washed with hot water. It remained like this for several days until it was cured. This was a very common healing practice in Galicia [90], the Basque Country [43, 44, 46, 77, 91] and Andalusia [92, 93].

In contrast, in the Canary Islands dog bites were cured by rubbing the wounds, not with the dog’s hair, but with the blood of the animal that caused them [52], and in Telleriarte (Guipúzcoa), in the past, the dog in question was forced to lick the wound, since it was considered that “its tongue was the cleanest” [44].

## Discussion

### Therapeutic uses throughout history

It is interesting to consider the great variety of medical remedies based on the dog and the products derived from dogs throughout history. The Celts associated dogs with the goddess Sequana by giving them votive offerings in exchange for cures [94]. If we turn to classical Latin authors such as Pliny the Elder (AD 23–79) we can observe dog-based remedies to treat earache, fevers and wound healing using their saliva [95]. In general, the therapeutic use of canids such as foxes, wolves and dogs stretched from the Greco-Roman world to the Middle Ages, as we can see in the literature of the fourteenth century, for example in the synthesis work entitled *Libro de las utilidades de los animales* (Book on the uses of animals) [96] or in more particular and specific books such as *Tresor de Beutat* (Treasure of Beauty), written in Catalan and containing medical and cosmetic recipes exclusively for women [97]. The Anglo-Saxon spell “mix dog urine with the blood of a mouse and slather on warts, they will soon be gone” is more recent [98]. With these examples, we can state that the list of dog products that have been used throughout history is wide and very diverse: canine teeth, liver, spleen, fat, bones, eyes, blood, paws, tongue, urine or excrement, among others. In our review, focused on contemporary Spanish ethnomedicine (in the twentieth century), the number of body parts of the dog used as medicine is significantly lower (Table 1).

**Table 1:** Therapeutic uses of dogs (*Canis lupus familiaris*) and their body parts or derivative products in contemporary Spanish ethnomedicine

Part or product used	ICD-10 chapter^a^	Diseases or ailments treated^b^	Preparation (administration route)^c^	Remedy type^d^	Geographical location	Ref. No.
Whole animal	I	Pulmonary tuberculosis (•)	Cooked (IN)	EMP-CUR	Alto Aragón (Huesca)	[42]
			Slit open from one end to the other (EX)	MAG-CUR	San Sebastián (Guipúzcoa) and Extremadura	[43–45]
	I	Tuberculous meningitis (•)	Cooked (IN)	EMP-CUR	San Sebastián (Guipúzcoa)	[43, 46]
	I	Whooping cough	Without prepraration (TR)	MAG-CUR	Extremadura	[45]
	I	Warts	Without prepraration (TR)	MAG-CUR	Extremadura	[56]
	I	Tertian and quartan fever	Without prepraration (TR)	MAG-CUR	Guijo de Granadilla (Cáceres), Fuenteliante (Salamanca) and Reus (Tarragona)	[57–59]
	III	Anaemia (•)	Cooked (IN)	EMP-CUR	Doñana (Andalusia)	[62]
	IV	Rickets (•)	Cooked (IN)	EMP-CUR	Asturias and provinces of Tarragona, Badajoz and Murcia	[57, 59, 63–66]
	X	Pneumonia (•)	Slit open from one end to the other (EX)	MAG-CUR	San Sebastián (Guipúzcoa)	[43, 44]
	X	Asthma (•)	Cooked (IN)	EMP-CUR	Fuerteventura (Canary Islands)	[69]
	XI	Stomatitis	Without prepraration (TR)	MAG-CUR	Asturias	[64, 65, 70–72]
	XI	Hernias in children (umbilical and inguinal)	Without prepraration (TR)	MAG-CUR	Aldeanueva del Camino (Cáceres)	[73]
	XII	Hangnails	Without prepraration (TR)	MAG-CUR	San Vicente de Alcántara (Badajoz)	[56]
	XII	Painful spots in breastfeds (•)	Cooked (IN)	EMP-CUR	Madroñera (Cáceres)	[76]
	XV	To obtain the correct shape of the nipples (•)	Without prepraration (EX)	EMP-PRE	Doñana (Andalusia) and the Basque Country	[44, 46, 62, 78]
	XV	Mastitis (•)	Without prepraration (EX)	EMP-CUR	Province of Soria and Extremadura	[40, 74, 80]
	XV	Milk stasis (•)	Without prepraration (EX)	EMP-CUR	Provinces of Navarra, Segovia and Soria, Treviño (Burgos), Cerdanya (Catalonia), La Alcarria (Guadalajara) and the Basque Country	[44, 74, 81–85]
	XV	Excess of milk (•)	Without prepraration (EX)	EMP-CUR	Basque Country and Navarra	[44, 78, 86, 87]
	XVIII	Jaundice	Without prepraration (TR)	MAG-CUR	Provinces of Alicante, Valencia and Murcia	[66, 88]
	XIX	Infected wounds (•)	Fried in olive oil (EX)	EMP-CUR	Ubierna (Burgos)	[89]
Saliva	I	Warts	Without prepraration (EX)	EMP-CUR	Lanzarote (Canary Islands)	[52]
	IX	Haemorrhoids	Without prepraration (EX)	EMP-CUR	Valencia del Ventoso (Badajoz)	[68]
	XII	Chapped lips	Without prepraration (EX)	EMP-CUR	Holguera (Cáceres), Zafra and Peloche (Badajoz)	[51]
	XII	Urticaria	Without prepraration (EX)	EMP-CUR	Santa Cruz de Paniagua, El Bronco and Palomero (Cáceres)	[75]
	XV	Fissures, sores and excoriations on the breasts	Without prepraration (EX)	EMP-CUR	Moraleja and Casas de Don Gómez (Cáceres)	[79]
	XIX	Superficial wounds (and cuts and sores)	Without prepraration (EX)	EMP-CUR	Provinces of Navarra, Salamanca and Soria, and Extremadura and the Basque Country	[41, 44, 57, 60, 68, 74, 79, 80]
	XIX	Chilblains	Without prepraration (EX)	EMP-CUR	Aldeanueva de Figueroa (Salamanca)	[41]
	XX	Insect bites and stings	Without prepraration (EX)	EMP-CUR	Morasverdes (Salamanca)	[41]
	XX	Viper bites	Without prepraration (EX)	EMP-CUR	Guijuelo (Salamanca)	[41]
	XX	Dog bites	Without prepraration (EX)	EMP-CUR	Telleriarte (Guipúzcoa)	[44]
Faeces	I	Diphtheria	Poultice (EX)	EMP-CUR	Ripollés (Catalonia)	[48]
	IV	Malnutrition	Boiled (IN)	EMP-CUR	Sierra de Segura (Albacete)	[53, 54]
	X	Colds	Tisane with different components (IN)	EMP-CUR	Sierra de Segura (Albacete)	[53, 54]
	X	Sore throat	Without prepraration (EX)	EMP-CUR	Ripollés (Catalonia)	[48]
	X	Pneumonia	Poultice (EX)	EMP-CUR	Ripollés (Catalonia)	[48]
	XI	Diarrhoea	Boiled (IN)	EMP-CUR	Bretún (Soria)	[74]
	XV	Weaning	Without prepraration (EX)	EMP-CUR	Basque Country	[86]
	XVIII	Epistaxis	Without prepraration (EX)	EMP-CUR	Arcos de Jalón (Soria)	[74]
	XX	Spider bites	Powdered (IN)	EMP-CUR	Canary Islands	[52]
Milk	I	Whooping cough	Boiled (IN)	EMP-CUR	Provinces of Cáceres and Salamanca	[41, 49, 50]
	IV	Rickets	Boiled (IN)	EMP-CUR	Provinces of Cáceres and Salamanca	[41, 49, 63]
	V	Alcoholism	Boiled (IN)	EMP-CUR	Alburquerque (Badajoz)	[57]
	VIII	Earache in children	Without prepraration (EX)	EMP-CUR	Líjar (Almería)	[67]
	X	Bronchitis	Boiled (IN)	EMP-CUR	Valencia de Alcántara (Cáceres)	[50]
	X	Colds	Boiled (IN)	EMP-CUR	Valencia de Alcántara (Cáceres)	[50]
	XV	Low milk supply	Boiled (IN)	EMP-CUR	Torrejoncillo (Cáceres)	[76]
Skin	II	Skin cancer	Burnt, ashes (EX)	EMP-CUR	Fuentes de León (Badajoz)	[51]
	VII	Cataracts	Without prepraration (EX)	MAG-CUR	Dehesa de Coquilla (Salamanca)	[58]
	XIII	Sciatica	Tanned by hand (EX)	MAG-CUR	Mountains of Álava province	[46, 77]
	XIII	Nocturnal leg cramps	Tanned by hand (EX)	MAG-PRE	Provinces of Álava and Navarra	[44, 60]
Fur	I	Rabies	Without prepraration (EX)	MAG-PRE	Oliva de la Frontera (Badajoz)	[51]
			Burnt, ashes (EX)	MAG-PRE	Canary Islands	[52]
			Fried in olive oil (EX)	EMP-PRE	Castile-La Mancha	[53, 54]
	XX	Dog bites	Fried in olive oil (EX)	EMP-CUR	Galicia, the Basque Country and Andalusia	[43, 44, 46, 77, 90–93]
Meat	II	Cancer (•)	Cooked (IN)	EMP-CUR	Abadiano (Vizcaya)	[44]
	V	Tantrum (without breathing, very red) (•)	Fried (IN)	EMP-CUR	Galisteo (Cáceres)	[58]
	VI	Hemiplegia (•)	Cooked (IN)	EMP-CUR	Villarino de los Aires (Salamanca)	[41]
Head	II	Skin cancer	Burnt, ashes (EX)	EMP-CUR	Galicia	[61]
Teeth	XI	Teething	Without prepraration (EX)	MAG-PRE	Province of Salamanca	[38, 41]
Claws	I	Rabies	Without prepraration (EX)	MAG-PRE	Province of Badajoz and Galicia	[51, 55]
Testicles	I	Diphtheria	Without prepraration (TR)	MAG-CUR	Extremadura	[47]
Sperm	I	Warts	Without prepraration (EX)	EMP-CUR	San Vicente de Alcántara (Badajoz)	[56]
Blood	XX	Dog bites	Without prepraration (EX)	EMP-CUR	Canary Islands	[52]

^a^ICD-10 chapter: I = certain infectious and parasitic diseases; II = neoplasms; III = diseases of the blood and blood-forming organs and certain disorders involving the immune mechanism; IV = endocrine, nutritional and metabolic diseases; V = mental and behavioural disorders; VI = diseases of the nervous system; VII = diseases of the eye and adnexa; VIII = diseases of the ear and mastoid process; IX = diseases of the circulatory system; X = diseases of the respiratory system; XI = diseases of the digestive system; XII = diseases of the skin and subcutaneous tissue; XIII = diseases of the musculoskeletal system and connective tissue; XV = pregnancy, childbirth and the puerperium; XVIII = symptoms, signs and abnormal clinical and laboratory findings, not elsewhere classified; XIX = injury, poisoning and certain other consequences of external causes; XX = external causes of morbidity and mortality

^b^(•) folk remedies based on the use of puppies

^c^Administration route: EX = external use; IN = internal use; TR = transference ritual

^d^Remedy type: EMP = empirical; MAG = magical / PRE = preventive healthcare; CUR = curative care

As is the case for other animal-based remedies, the use of the dog in Spanish ethnomedicine has a philosophical basis with a strong influence of humoralism. In the subsequent work that we are conducting it can be seen that zootherapeutic traditional knowledge has a historic origin, based on the doctrine of “Greek humoral pathology”, magical thinking and the ideological-symbolic subsystem of the rural world [11, 41]. Hippocratic medicine, by way of humoral theory, explained the physiology of the body by the balance of the four humours: black bile (cold and dry), yellow bile (hot and dry), phlgm (cold and moist) and blood (hot and moist). The excess or deficiency of these four basic substances, as a consequence of lifestyle, would cause ailments, disorders or diseases that would have to be countered in order to regain health. Therefore, humoral therapy is based on treatment by opposites, following the principle of *contraria contrariis curantur* [99, 100].

In the twelfth century, Hildegard of Bingen (1098–1179), saint and author of medical tracts, in applying the humoral theory ascribed a “very hot” character to the dog [101]. According to this theory, its medicinal properties were related to bile and the warmth of its tongue soothed wounds and ulcers very effectively. However, she did not consider the other parts of the dog as good therapeutic resources, and suggested that they were not useful for medicine [101]. Abu-S-Salt Umayya (1068–1134) also defended the very hot character of this domestic animal. Specifically, this Andalusian polymath describes the excrement of dogs as “a hot and soft medication, an essence, like gum ammoniac”. In his work *Treatise on simple medicines*, which was translated into Latin by Arnau de Vilanova in Valencia in around 1280, he includes it in the “simple medicines that exert general actions on the whole of the body, without being specific to a particular organ” [102]. In *Lilium medicinae*, printed in Naples in 1480, Lyon in 1491, and Venice in 1494, Bernard de Gordon (fl. 1270–1330) notes the therapeutic effects of the excrement of persons and of animals on certain conditions. So, for the treatment of *squinancy* or quinsy (peritonsillar abscess, a complication of tonsillitis), he declares: “the faeces of a dog that gnawed bones were very useful”. The faeces had to be dried on a tile, reduced to a powder and cooked with mead, and this mixture had to be gargled [103].

It is worth noting that the remedies described above remained clearly in use until the end of the twentieth century, and that their status was official and university approved in the seventeenth and particularly the eighteenth century, known as the “Century of Enlightenment”. So, concerning the subject of white dog excrement, in the seventeenth century the apothecarist Francisco Vélez de Arciniega published in Madrid his extensive work entitled *Historia de los animales mas recebidos en el uso de la Medicina* (History of the animals most widely used in medicine), where he writes: “Make use of the dung of dogs, which have eaten only bones for two days (according to Galen), dry and ground up the white part, for *squinancy*, dysentery and very old sores” [104]. And in the eighteenth century official and university publications appeared with recipes containing white excrement of dogs as the main ingredient [105].

On the other hand, as an interesting fact that speaks of the implementation of the theoretical-practical system of humoral medicine outside of the European geographical and cultural context, we cite the Portuguese doctor Simão Pinheiro Morão (*c*. 1618–1685). He practised medicine in the late seventeenth century in the Pernambuco, Brazil. He was a steadfast defender of humoralism given his training in Salamanca and Coimbra, universities that at the time were hegemonic and attached to humoralist doctrine. Morão, like other Old World doctors, assimilated tropical diseases to the European academic nosology and belittled indigenous and Afro-Brazilian remedies and healing practices, which he considered “empirical” [106]. Among zootherapeutic prescriptions – from Europe or Asia – that Morão prescribes including dogs [107], there is mention of the placement of a newborn puppy, slit open from one end to the other and still warm, on the head of a “maniac” patient. Furthermore, to treat epilepsy, Morão adopts the advice of the German doctor Mathias Untzer regarding the use of bile from breastfed black puppies. The drops of bile must be drunk with cherry water and the puppies must be killed by drowning. Male patients require male puppies, while female patients require female puppies [106].

Another classic work from the mid-eighteenth century (1751) is the manuscript *Libro de medicinas, muy seguro, para curar varias dolencias, con yerbas muy experimentadas, y provechosas, de esta provincia de Yucathan* (Book of Medicines, very safe to treat various illnesses with well proved and useful plants from this province of Yucatán), about traditional Mayan medicine from Yucatán (Yucatán Peninsula, Mexico). In this book, despite the dominant appearance of purely indigenous concepts and remedies (including both animals and plants), some of European origin are included. The influence of traditional European medicine is clear, for instance in recipes that prescribe the “use of […] ox or dog excrement, […]” [108]. Curiously, a recipe to cure cancer attributed to St. Albert the Great appears: in it, the head of black dog must be burnt and then the ashes spread on the cancerous area [108]. Also, in *The Book of Chilam Balam of Ixil* (neither its place of provenance nor exact date are known), written in the Yucatec Maya language and with subtitles in Castilian Spanish, the “use of a drink prepared with ram tallow and burnt excrement of dog” to treat diarrhoea is mentioned [109]. Moreover, in order to treat spleen pain, *The Book of Chilam Balam of Kaua* (18th century) suggests “placing on top the same organ, previously heated, from a dog” (which itself was thought to be “hot”) [110]. Despite this Spanish and European influence in the traditional medicine systems of indigenous peoples who, like the Yucatec Maya, still inhabit the Mesoamerican region, there remains concrete evidence of the continued use of the dog in medicine from pre-Hispanic times. Clear proof of the fact that Nahua medicine was indeed holistic can be found in the codex *Libellus de Medicinalibus Indorum Herbis* (Treatise on indigenous medicinal herbs), also known as the *De la Cruz-Badiano Codex* (1552). More than a herbal book, it includes data on the combined use of minerals and animals (between 60 and 88 species) in several healing recipes [111, 112]. Three treatments against hair loss, dandruff prevention and against armpit odour involving dogs – urine, bile and bones, respectively – as an ingredient.

### Traditional medicinal practices

A total of 63 popular remedies to treat and/or prevent as many as 49 human diseases or ailments have been inventoried; of these, 46 are empirical and 17 magical. The numerical majority of these remedies are for the treatment of certain infectious diseases (Chapter I of the ICD-10), all in all, these sum 15 remedies. On the other hand, those associated with the postpartum period (Chapter XV), those for the treatment of respiratory diseases (Chapter X), and those for treating or alleviating animal bites and stings (Chapter XX) follow in importance, with six or seven remedies each (see Table 1).

In 20 of the documented remedies the whole animal was used; the use of puppies or litters of pups was recommended in 12 cases. In five of these, it was also stressed that new-born puppies should be used. We also found that saliva is the healing element in 10 remedies, in general based on offering the injured area to a dog so that it can lick it. Excrement is the therapeutic basis of nine remedies and the milk of a bitch of seven. The use of skin, fur, and meat follows in importance (see Table 1). Regarding the meat, in two out of three remedies in which the consumption of dog meat was recommended, the advisability of ingesting puppy meat is also emphasised.

Undoubtedly, the most relevant dog-based therapeutic resource in the Spanish ethnomedicine is the use of the whole animal. The medical use of that resource appears registered in 33 of the requested products, followed in importance by fur, saliva, and milk cited in 12, 11, and seven works, respectively. Of scarcer appearance are the use of skin and faeces, cited in six references, as well as that of meat, claws, and teeth cited only twice or thrice. Dog blood, sperm, head, and testicles appear collected only in a single bibliographic reference each. This approach provides information on the relative importance of different parts or secretions used as therapeutic resources in general. However, it is interesting note that whole animals, feces, and fur are the products which are most recorded for the treatment of a particular group of pathologies. Thus, whole animals are the most used resource in the treatment of infectious diseases and conditions related to pregnancy, childbirth, and the puerperium (Chapters I and XV). Meanwhile, dog saliva is used for wound cleaning and surface treatment (Chapter XIX) and dog fur is mainly employed for dog bites (Chapter XX). Although these data cannot be analyzed statistically, they provide reliable quantitative information about the most used dog parts or secretions. We can state that in Spain other dog-based therapeutic elements might be vestigial resources from the early twentieth century, mainly, such is the case of blood, sperm, head, and testicles.

It is important to note that, the fact that these remedies have endured over time–even those that are now rarities–points to the relevance of continuing to study, catalogue, and inventory this knowledge from an ethnobiological point of view. This is especially so in the case of wild animals that require significant tasks of taxonomical identification integrating historical, biogeographical, and biological methods that allow data clarification for vernacular names contained in the literature. Regarding domestic animals, such as the one at hand, usage data collected in future research can help to understand diseases and their relation to traditional therapeutic resources. Evidently, ethnomedicine is not ahistorical, but rather a social construct; therefore, the fact that many documented zootherapeutic resources were still in use until mid-twentieth century in a society like that of Spain is a subject for thought and historical-philosophical debate. This review, together with another that was recently conducted, constitutes a basis for future studies which can be profitable efforts for taxonomical identification, while also determining the cultural importance that these folk remedies had or still have. Likewise, it also offers a critical approach to discuss a very interesting matter for the Spanish epistemological ethnozoology: what qualities do certain animal-based remedies have for them to continue being used in an increasingly medicalized society?

In this review the importance of the colour of the dog is not significant, we have only documented one remedy based on the use of one white dog and three black dogs. This could be a reflection of the ambivalence of Spanish TK, since in popular Spanish superstition black is the colour associated with death and bad luck [11, 41, 57, 113], and white is the complete opposite. However, white faeces may be a symptom of gastrointestinal problems in an animal and, consequently, its use has to do with homeopathy: “evil to treat evil”. On two other occasions another homeopathic remedy is mentioned: the use of certain parts obtained from a rabid dog to treat this same infectious disease transmitted by dogs.

Outside of Spain, other studies – which are non-specific regarding the colour of the dog – also report the use of dog faeces. For instance, in southern Italy, faeces are topically applied to treat skin burns [54, 114], while in the Eastern Rraicë and Mokra areas (Eastern Albania) they were used to cure hepatitis [115]. In this second case, faeces were mixed with flowers and baked into a small loaf, then given to the affected person to eat; the affected person was not supposed to know about the exact nature of the bread. Ceríaco reports the use of toasted and ground dog faeces as a haemostatic on wounds in Portugal [116]. Across the Atlantic, studies from north-eastern Brazil are prominent in that they have found dog faeces to be a versatile part of the ethnomedicine. They aid in the treatment of an ample range of ailments such as asthma, measles, chickenpox, mumps, smallpox and menstrual cramps [117–122]. In general, faeces are diluted and drunk as an infusion (“faeces tea”), although Costa-Neto [118] and Costa-Neto and Oliveira [119] mention, respectively for measles and chickenpox, that faeces are dried and then boiled (inside a handkerchief); afterwards, the resulting paste is spread on the red spots on the skin. That is, the same procedure is followed to treat different diseases when they both manifest through exanthema.

As is the case with faeces, the relevance awarded to saliva – it appears in 10 remedies – makes it one of the main curative elements obtained from dogs. Its use is recorded within the Albanian communities living in Italy [54, 123] and Serbia [124]. In the first case it was used to treat boils (no longer used), while for Albanians inhabiting the Pešter plateau (south-western Serbia) it was used to remove warts; a young dog had to lick them. In Ireland an ancient cure for chapped skin, bunions and burns was to get a dog to lick the affected part [125]. In Portugal Ceríaco also reports the use of both dog’s saliva and urine as antiseptic and haemostatic agents [116]. From the ethnopharmacological point of view it should be noted that scientific evidence exists concerning the antimicrobial and anti-inflammatory activity of dog saliva [126, 127], even at low concentrations [128].

The widespread practice of using dog hair to heal wounds and to avoid rabies infection in Spain when someone was bitten has also been reported in studies carried out in other European countries: Albania [54, 129], Italy [54, 130] and Portugal [116]. Furthermore, according to DuBois and Lang [131] Sami people (from northern Sweden) in the early twentieth century used to rub the wound caused by a dog bite with blood from the dog. On the other hand, in Transylvania (Romania) three traditional remedies including dog hair are reported: a) smoked hairs to treat dog bites, b) burnt hairs mixed in wine for toothache, and c) the smoke obtained from hairs placed on a shovel and put in the stove for one night breathed in against madness [132].

The use of both dog meat and fat has been reported by Sõukand and Pieroni [133] among the Ukrainian inhabitants of the transfrontier region of Bukovina to treat tuberculosis. Furthermore, the authors mention the use of fat from dogs mixed with vodka and rubbed on the chest to treat colds. At the opposite extreme of Europe, in Portugal, people ate the head or the heart of the dog to heal rabid dog bites [116]. Additionally, this kind of use is found in some Mexican indigenous groups; for instance, the Tlahuica community from the municipality of Ocuilan (Mexico State) treats patients affected by typhoid fever by binding the meat of a small black dog to their bodies [134].

However, not only do we find coincidences in the empirical type of dog-based remedies, magical remedies are also documented in other countries. In Meãs do Campo (Coimbra district, Portugal) the following remedy was practised on St. John’s Eve to remove warts: warts were counted and the same number of grains of salt were put inside a piece of bread that was given to a dog to eat, leaving the person free of his/her warts [135].

Also in Portugal, it was a very well-rooted custom to carry a dog’s tooth in a pocket or hung around the neck to prevent toothache [116, 135], as well as the use of some rituals of transference. Thus, to combat fever the patient’s nails were placed in bread and given to a dog to eat, and as an anti-madness ritual, a dog or a pup was opened up and placed on the mad person’s head so the dog’s blood covered his/her face [116]. For their part, at the northern tip of Europe, Sami people passed partially chewed food to a female dog to avoid pregnancy cravings, and believed that the simple presence of a dog drew illness away [131].

It is interesting to note that Christian iconography, hagiography and symbolism have reinforced many beliefs to do with healing. St. Roch is one of the saints associated with folk medicine in Spain [41, 44, 77, 94], and he is frequently shown in images with a dog licking his wounds to heal them [136]. Perhaps this iconography is a Christianisation of the Celtic goddess Sequana mentioned earlier, since she is also often shown accompanied by a dog [94]. Christian art places the dog as a symbol of happiness and represents it as a guardian and defender of herds, and a friend devoted to man; it is therefore an animal associated with well-being [136–138]. Evil and disease are fought using a symbol of good; a good example is the treatment of all the respiratory conditions [41, 45, 49, 50]. Secondly, we may think of a therapeutic product that is very widely used such as milk, which has a Eucharistic meaning and represents eternal happiness, as well as demonstrating symbolism relating to wisdom, purity and renewal in spring [137, 139]. It is not surprising that bitch’s milk should have featured in rituals of the transfer of evil and been qualified as “something holy” for earache [67]. Likewise, the empirical remedy of “bitch’s milk” is interesting, since its use coincides with the use of donkey’s milk against rickets [10].

On the other hand, it is worth noting that only one datum was found on a specific dog breed. This was the *bardino* or *majorero* dog, a breed of cattle dog which had been living all over the Canary Islands since ancient times, and a rustic, hard-working breed with a fiery character [140]. This character, more than might be expected in other breeds, meant that according to humoral theory, a remedy prepared from it against asthma would be considered very effective [69].

From the ethical aspect, the use of dogs is generating much discussion with regard to animal rights. Some of the benefits that certain cultures obtain from these animals are very controversial and are currently widely rejected. Well-known examples include the consumption of dog meat in South Korea and the industry derived from it [141] or the celebration of the annual Dog Meat Festival in Yulin (China) [142]; but, even in Switzerland dogs have regularly been eaten by farmers in rural areas [143]. There is no doubt that some Spanish medical practices of the early twentieth century would meet with this type of rejection, a time in history when dog meat was eaten, for example, to treat cancer or hemiplegia [41, 44].

In contrast to these ethically rejected medical uses of dogs there is AAT. In Spain these practices developed from the end of the last century, as a result of the efforts of various organisations such as the Fundación Affinity (http://www.fundacion-affinity.org/) or the ONCE Guide Dog Foundation (http: //perrosguia.once.es/es). Since then, pet therapy, in particular the use of therapy dogs, has spread widely and is on the rise [144–146]. Its effectiveness is based on the psychological, emotional, playful aspects and physical stimulation that occur in the interaction between humans and canids [20, 147].

Obviously, folk medicine and AAT are two therapeutic options that arise within concrete and very different cultural contexts, responding, in both cases, to social needs at a particular time in history. So, when referring to ways of understanding and attending to health and well-being, the word “ethnomedicine” would mean to us systems of healthcare created in a particular human group to meet the health needs of its members and to help maintain the cohesion of that community within a shared group project, within its own traditions. The remedies based on the use of dogs are not part of Spanish ethnomedicine at present and, to consider them as such would be ahistorical. However, we can state that the ethnomedical use of dogs does exist and has been transferred to AAT.

## Conclusions

The idea of health and well-being of Spanish society today has gradually moved away from the use of traditional animal-based remedies. However, it is a documented fact that the dog has been a very important therapeutic resource throughout history. The historical depth of the knowledge and handing down of its medicinal use led to it becoming a versatile traditional remedy until its extinction in the twentieth century. The ways of understanding and providing healthcare are human creations that, as they respond to the needs of societies and cultures, develop in a different manner in each one, always within the bounds of tradition and custom. All human groups follow their own lines of development inherited from a collective past. Consequently, cultures are constantly being created and destroyed, reflected in their historic presents. In the case of the dog, the therapeutic uses of the twentieth century have been replaced by ethnomedical uses centred around AAT. Dogs continue to play a role in health and to form an important part of non-material culture. All of this will translate into beliefs and ways of symbolic thinking about this domestic animal, some based in folk medicine and ancient traditions and with a certain amount of transformation and evolution, or perhaps other new ones arise as a consequence of a new scenario. Nevertheless, it is important to emphasise that the current use of dogs in Spanish ethnomedicine is not the result of a slow and gradual accumulation of TK, but rather that of a “paradigm shift”. This has come about from a new way of thinking and understanding about health and therapeutic resources in Spanish society, which has only left a place for dogs there in the area of AAT. On the other hand, this review invites epistemological reflection on the historical, anthropological and ethical contributions of the zootherapeutic resources in Spain. From the perspective of Spanish ethnozoology, it is necessary to make an inventory and checklist of species (wild and domestic) and medicinal uses; however, once this objective has been achieved, some reflection is needed on the relationship of human beings to the remedies. The data produced contribute to this objective.

Finally, we hope that this work may be useful for future studies of ethnomedicine in non-European contexts, especially in areas such as Latin America, where indigenous elements, classical medicine and Spanish and Portuguese folklore, as well as an African influence, have created a body of knowledge of great cultural richness.
